# Anterior cervical discectomy and fusion in treating acute myelopathy caused by *Brucella* cervical spondylitis: a case series

**DOI:** 10.1080/07853890.2025.2493308

**Published:** 2025-04-18

**Authors:** Long Chen, Tao Zhang, Xing-yu Chen, Yi-zhe Wang, Xing-guo Tan, Da-shuai Huang, Yan-peng Lu, Song-kai Li

**Affiliations:** ^a^ Department of Spinal Surgery, the 940th Hospital of the Joint Logistic Support Force of Chinese PLA, P. R. China; ^b^ First Clinical Medical School, Gansu University of Chinese Medicine, Lanzhou Gansu, P. R. China; ^c^ Department of Surgery, Sichuan Provincial People’s Hospital, Chengdu Sichuan, P. R. China

**Keywords:** *Brucellar* cervical spondylitis, spinal cord impairment, clinical presentation, ACDF surgery, clinical benefit

## Abstract

**Background and Objectives:**

Cervical disease caused by *Brucella* infection is rare, with acute spinal cord impairment due to myelitis being a severe complication. If untreated, it can lead to significant patient damage. This study aims to begin to investigate the clinical characteristics of acute cervical myelitis caused by *Brucella* infection and to evaluate the profile of clinical benefit of anterior cervical discectomy and fusion (ACDF) for this condition.

**Methods:**

This retrospective case series included 6 patients who underwent surgical treatment for acute cervical spinal cord impairment due to *Brucella* infection at our institution between January 1, 2013, and January 1, 2023. Clinical data such as age, gender, surgery duration, follow-up period, medication duration, time to bone fusion, ASIA classification, Visual Analog Scale (VAS) score, Japanese Orthopaedic Association (JOA) score, and Neck Disability Index (NDI) score were collected both preoperatively and postoperatively. Statistical analysis was used to assess the clinical benefits of ACDF surgery.

**Results:**

Six patients (4 males, 2 females) underwent successful ACDF. The median age was 52.5 years. The median surgery duration was 130.0 min, and the median hospital stay was 13.5 days. The median follow-up period was 15.0 months, and the median duration of postoperative medication was 16.0 weeks. All patients achieved satisfactory bone graft fusion, with a median fusion time of 4.0 months. ASIA classifications improved in all patients. Three patients improved from grade C to grade E, two from grade B to grades D and E, and one from grade C to grade D. The median VAS score decreased from 6.0 preoperatively to 0.0 at follow-up. The median JOA score increased from 6.0 to 17.0, and the NDI score improved from 29.5 to 4.5. No recurrence of infection or neurological symptoms was observed during follow-up.

**Conclusion:**

Acute cervical spinal cord impairment from *Brucella* infection is rare and challenging to diagnose early. However, early ACDF application effectively relieved spinal cord compression, improved neurological symptoms, and enhanced patient outcomes, demonstrating its efficacy for treating acute myelitis caused by *Brucella* infection.

## Introduction

Since the discovery of *Brucella* species, significant progress has been made in understanding their biological characteristics, pathogenic mechanisms, and the harm they inflict on both humans and animals. *Brucellosis* remains a pressing public health issue, particularly prevalent in developing countries with strong agricultural and animal husbandry sectors. It imposes a substantial burden on local populations and the socioeconomic system [1–3]. Worldwide, there are over 500,000 new cases of *brucellosis* each year, with significant regional variations in incidence rates [4]. In China, *brucellosis* is primarily concentrated in regions with developed animal husbandry, such as the Qinghai-Tibet Plateau, northwest, and northeast areas [2,5,6]. The incidence rate has shown a gradual increase in recent years, rising from 0.92 per 100,000 population in 2004 to 4.2 per 100,000 in 2014. Following a brief decline, the incidence rate began to rise steadily again in 2017, reaching 4.95 per 100,000 by 2021 [5,7,8]. Among the severe complications of *brucellosis* is *Brucella* spondylitis [2,9]. Current research indicates that approximately 70% of *Brucella* spondylitis cases involve the lumbosacral region, about 20% affect the thoracic vertebrae, and less than 10% involve the cervical spine [10,11]. Acute spinal cord injury resulting from cervical *Brucella* infection is rarer but can cause irreversible and severe damage when it occurs [11,12].

Due to the rarity of cervical *Brucella* infections, no definitive treatment guidelines have been established. The existing literature generally supports conservative medical management, which involves a triple-drug regimen of doxycycline, rifampicin, and streptomycin, as recommended by the World Health Organization (WHO) [13,14]. However, some studies suggest that surgical intervention may be combined with conservative treatment for cases with spinal instability, prevertebral abscesses, or neurological symptoms, demonstrating favourable outcomes [11,12, 15–18]. Cervical spinal cord injuries typically have a poor prognosis, but early surgical intervention for most traumatic spinal cord injuries can significantly improve prognosis and quality of life [19,20]. Nevertheless, there is a lack of research on the efficacy of ACDF for acute spinal cord impairment caused by cervical *Brucella* infections. Therefore, this study aims to explore the therapeutic effects and clinical benefits of early ACDF surgery in patients with acute spinal cord impairment due to cervical *Brucella* infection through a series of case reports.

## Methods

### Study objective

This study aims to explore the effect of ACDF on the clinical outcome of acute spinal cord impairment caused by cervical *brucella* infection.

### Study design

A retrospective analysis was performed, incorporating clinical data from patients who underwent anterior cervical discectomy and fusion (ACDF) surgery at our institution between January 1, 2013, and January 1, 2023, for acute spinal cord impairment caused by cervical *Brucella* infection. The study was approved by the ethical review Committee of the 940th Hospital of the Joint Logistics Support Force of the Chinese People’s Liberation Army (approval number: 2023KYLL042), and the data were anonymized, which waived the requirement for informed consent. The diagnosis of *Brucella* infection was based on clinical evaluation and serological tests, including the *Brucella* Standard Tube Agglutination Test (STAT) and the Rose Bengal Plate Test (RBPT). Diagnostic criteria included a STAT titer of 1:100 (a fourfold increase) or a positive RBPT result, confirmed by the isolation of *Brucella* species from blood, tissue, or other body fluids [17,21]. The diagnostic standards were as follows: (1) a relevant history of epidemiological exposure; (2) clinical and imaging features consistent with cervical spondylitis, with other infections excluded; (3) positive *Brucella* culture or STAT positivity (at least a fourfold increase) or RBPT positivity. This study was designed to begin assessing the clinical benefits of ACDF surgery for the treatment of this condition. Therefore, the recovery of spinal cord injury (manifested by improvement in paralysis symptoms and ASIA grade) or a minimum follow-up of 12 months was designated as the endpoint for this cohort.

### Inclusion and exclusion criteria

**Figure 1.**
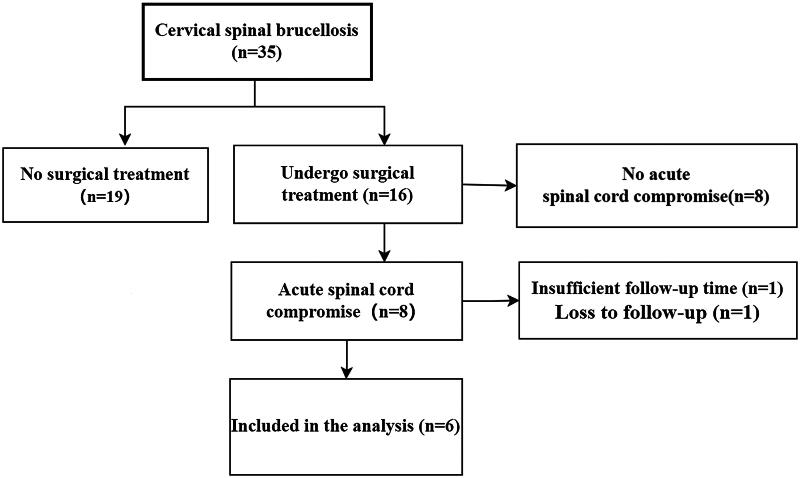
Flow chart of patient selection.

Inclusion criteria: (1) diagnosed with cervical *Brucellosis*; (2) occurrence of spinal cord impairment (Imaging evidence of spinal cord compression or invasion, accompanied by motor, sensory, and reflex changes); (3) treated with ACDF surgery; (4) available follow-up data for at least 12 months. Exclusion criteria: (1) presence of other infections; (2) absence of spinal cord impairment; (3) previous history of anterior cervical spine surgery; (4) loss to follow-up or incomplete follow-up data. (Figure 1)

### Patient management

A total of 35 patients with cervical *brucellosis* were admitted during the study period, of whom 16 underwent surgical treatment. Among those who received surgical intervention, 8 patients developed acute spinal cord impairment. Of these, 1 patient was lost to follow-up, and 1 had insufficient follow-up time. Ultimately, 6 patients met the inclusion criteria and were included in the study. All patients had their spinal cord impairment confirmed by magnetic resonance imaging (MRI) and underwent ACDF. Preoperative evaluations included X-rays and computed tomography (CT) scans to assess cervical vertebral destruction. Data on VAS scores, JOA scores, NDI scores, erythrocyte sedimentation rate (ESR), C-reactive protein (CRP), and white blood cell count (WBC) were recorded before surgery. During the procedure, pus and necrotic tissue samples were collected for biopsy and *Brucella* culture. The surgery was conducted by a team of spinal surgeons with extensive experience in ACDF procedures. Iliac bone was initially harvested as a graft, and the standard ACDF technique was followed. After the removal of purulent and necrotic tissue, the wound was irrigated with rifampicin solution (Figure 8). In two patients, the iliac bone was directly placed into the intervertebral space, while in the other patients, the iliac bone was crushed and inserted into the fusion cage before being placed in the intervertebral space. Rehabilitation, including limb exercises, was started on the second day after surgery. The intensity of rehabilitation was progressively increased based on the patients’ recovery, and patients were advised to continue rehabilitation, including limb function exercises, upon discharge. Postoperatively, patients were treated with rifampin (600 mg/day) and doxycycline (200 mg/day) [11,16,22]. The effectiveness of antimicrobial therapy was assessed by monitoring WBC, ESR, CRP, RBPT, and STAT levels. At the time of discharge, at the 3-month follow-up, and at least at the 12-month follow-up or the final follow-up, the patients’ VAS, JOA, and NDI scores were recorded to assess clinical efficacy and neurological function. Additionally, WBC, ESR, and CRP levels were measured at each follow-up visit to assist in evaluating clinical outcomes, detecting drug-related adverse effects, and identifying early signs of infection recurrence. X-rays and/or CT scans were conducted every 3 to 6 months postoperatively to assess bone graft fusion. Based on prior literature, the criteria set for the cessation of antibiotics are as follows [23,24]: (1) considerable alleviation of spinal pain and resolution of inflammatory responses; (2) normalization of body temperature; (3) normalization of CRP and ESR levels, with negative results on RBPT and STAT tests; (4) MRI demonstrating no signal alterations.

### Data analysis

Statistical analysis was performed using SPSS version 27.0 (IBM Corp., Armonk, NY, USA). Given the small sample size, inferential statistical tests were not conducted, and only descriptive analysis was performed. The median and interquartile range were used to statistically describe clinical data, including WBC, ESR, CRP, VAS, JOA, and NDI scores, at admission, discharge, 3-month follow-up, and final follow-up, in order to ensure the stability and reliability of the analysis results.

## Results

### Clinical characteristics

The cohort comprised four males and two females, with a median age of 52.5 years (interquartile range [IQR], 46.0–59.25 years). The median duration of surgery was 130.0 min (IQR, 120.0–156.25 min), and the median length of hospital stay was 13.5 days (IQR, 8.5–16.75 days). The median follow-up period was 15.0 months (IQR, 12.0–33.0 months), and the median duration of postoperative medication was 16.0 weeks (IQR, 12.0–21.0 weeks). According to the ASIA spinal cord injury classification, 2 patients were classified as grade B, and 4 as grade C. Severe clinical manifestations were noted in some patients, including one with severe fecal incontinence and another with severe urinary retention. All patients had a history of neck pain lasting more than one month (median: 1.5 months, IQR, 1–3.75 months). The median duration of paralysis symptoms caused by acute spinal cord injury was 3 days (IQR,1.75–7.75 days). All patients were from *Brucella*-endemic regions and had relevant exposure histories. Specifically, 5 patients were involved in the farming of cattle and sheep, and 1 patient had consumed unpasteurized milk from an endemic area before the onset of symptoms. Two patients had a history of fever, although blood cultures collected during fever episodes were negative. (Table 1)

**Table 1.** Basic clinical characteristics of the patients.

No	Gender/Age	Segment	Fever	Complications	Neck Pain(Months)	Paralysis Duration (days)	Surgery Duration (min)	Hospitalization Days	Graft Fusion Time (months)	Follow-up (months)	Blood Culture	Bacterial Culture	Risk Factors	Medication Method	Postoperative Medication Duration (weeks)	Iliac Crest Harvested?
1	M/50	C4-5/6-7	Yes	None	1	1	155	7	5	15	–	–	Animal Husbandry	Rifampin, Doxycycline	24	No
2	M/60	C4-5	No	Klippel-Feil Syndrome	1	2	160	9	4	84	Not Done	–	Unpasteurized Milk	Rifampin, Doxycycline	16	Yes
3	F/59	C6-7	No	None	2	2	120	25	3	12	Not Done	–	Animal Husbandry	Rifampin, Doxycycline	12	Yes
4	M/46	C5-6	Yes	None	1	4	130	13	4	16	–	–	Animal Husbandry	Rifampin, Doxycycline	16	No
5	M/46	C6-7	No	None	2	7	130	14	3	15	Not Done	–	Animal Husbandry	Rifampin, Doxycycline	20	Yes
6	F/55	C5-6	No	None	9	10	120	14	4	12	Not Done	–	Animal Husbandry	Rifampin, Doxycycline	12	Yes
M(IQR)	52.5 (46-59.25)				1.5(1-3.75)	3(1.75-7.75)	130(120-156.25)	15 (12-33)	4 (3-4.25)	13.5 (8.5-16.75)					16 (12-21)	

### Imaging findings

**Figure 2.**
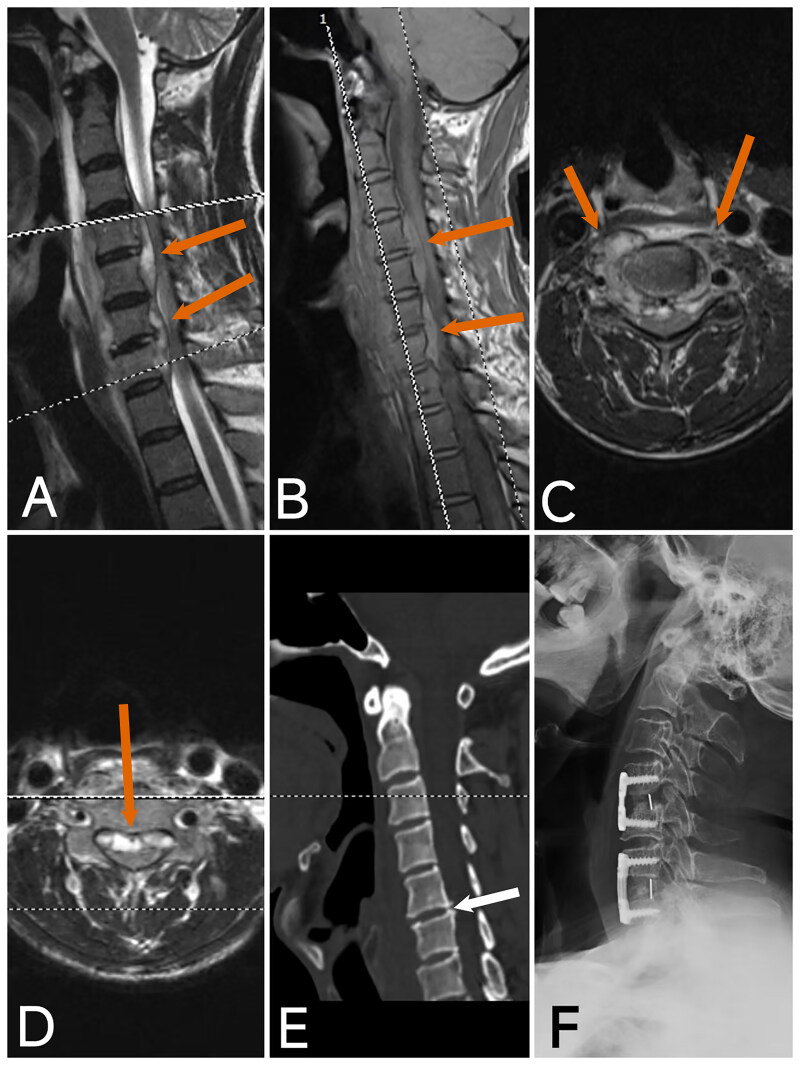
Image of case 1. A: T2-weighted imaging (T2WI); B: T1-weighted imaging (T1WI); shows a posterior epidural abscess in the spinal canal from C4 to C7, causing significant compression of the spinal cord (red arrow). C, D: Axial T2WI demonstrates epidural abscesses at two segments with notable compression of the spinal cord (red arrow). E: CT reveals cervical instability, loss of cervical curvature, loss of intervertebral disc height, and slight anterior displacement of C7 (white arrow). F: Postoperative X-ray at 5 months shows good positioning of the internal fixation, restoration of intervertebral height, recovery of cervical curvature, and intervertebral bone graft fusion.

**Figure 3.**
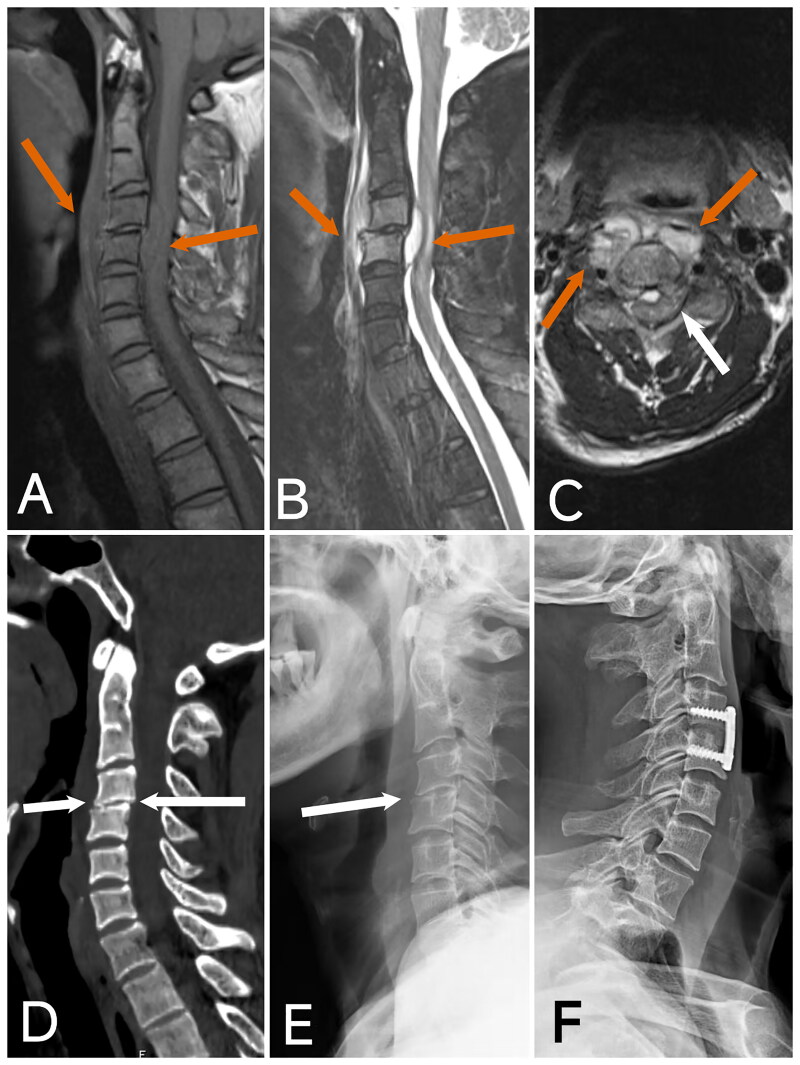
Image of case 2. A: T1-weighted imaging (T1WI); B: T2-weighted imaging with fat suppression (T2WI-FS); C: Axial T2WI shows a prevertebral abscess and epidural abscess with significant compression of the spinal cord, along with a linear area of high T1 and T2 signal within the spinal cord (red arrow). D, E: CT and X-ray reveal C2-3 segmental non-union, marked narrowing of the C4-5 intervertebral space, vertebral instability, slight posterior displacement of the C4 vertebra, and prevertebral bone destruction (white arrow). F: Postoperative X-ray at 4 months shows good positioning of the internal fixation, successful bone graft fusion, correction of the C4 posterior slip, restoration of cervical curvature, and recovery of intervertebral height.

**Figure 4.**
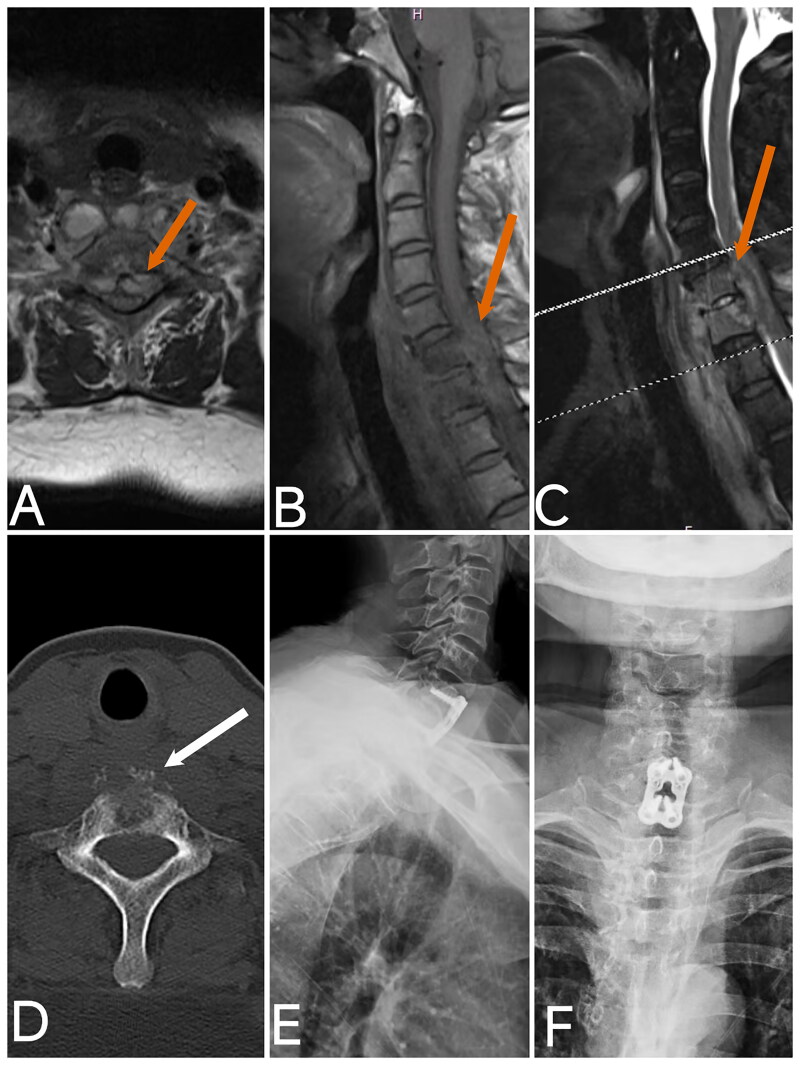
Image of case 3. A: Axial T2-weighted imaging (T2WI); B: T1-weighted imaging (T1WI); C: T2-weighted imaging with fat suppression (T2WI-FS) shows an epidural abscess with significant compression of the spinal cord (red arrow). D: CT reveals prevertebral bone destruction (white arrow). E, F: Postoperative X-ray at 3 months shows good positioning of the internal fixation.

**Figure 5.**
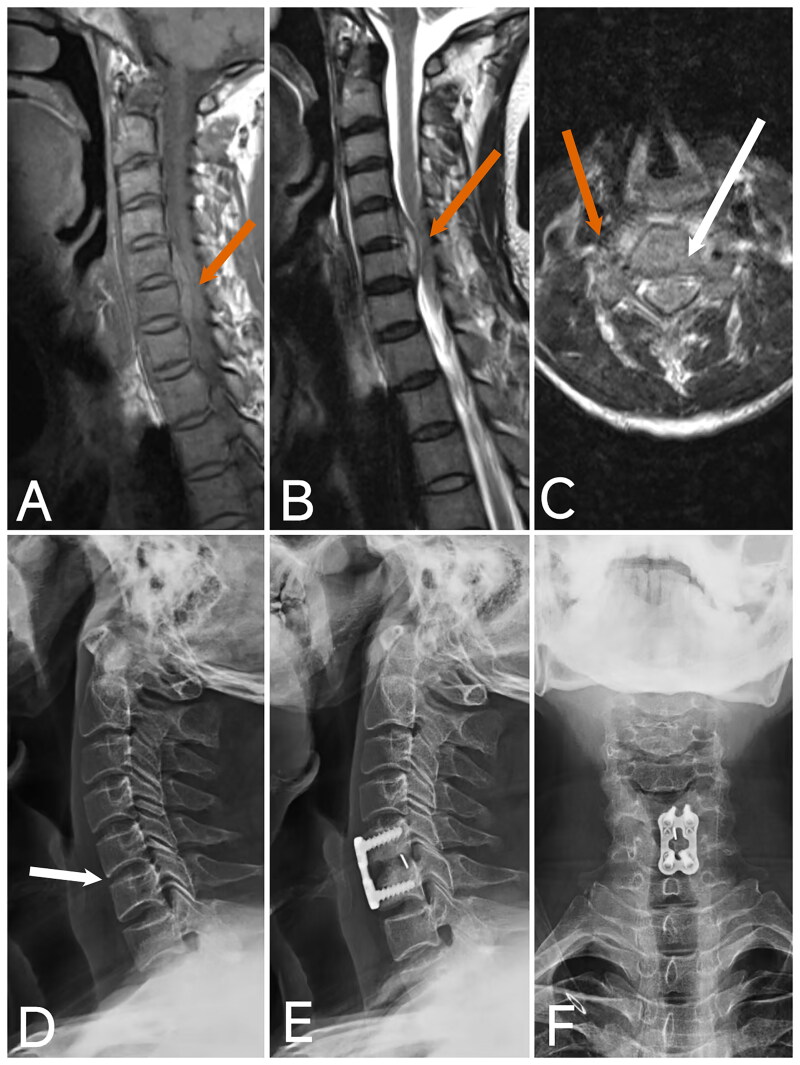
Image of case 4. A: T1-weighted imaging (T1WI); B: T2-weighted imaging (T2WI); C: Axial T2-weighted imaging (T2WI) shows an epidural abscess with significant compression of the spinal cord (red arrow). D: X-ray shows no significant bone destruction or loss of intervertebral height (white arrow). E, F: Postoperative X-ray at 4 months demonstrates good positioning of the internal fixation and fusion of the intervertebral bone graft.

**Figure 6.**
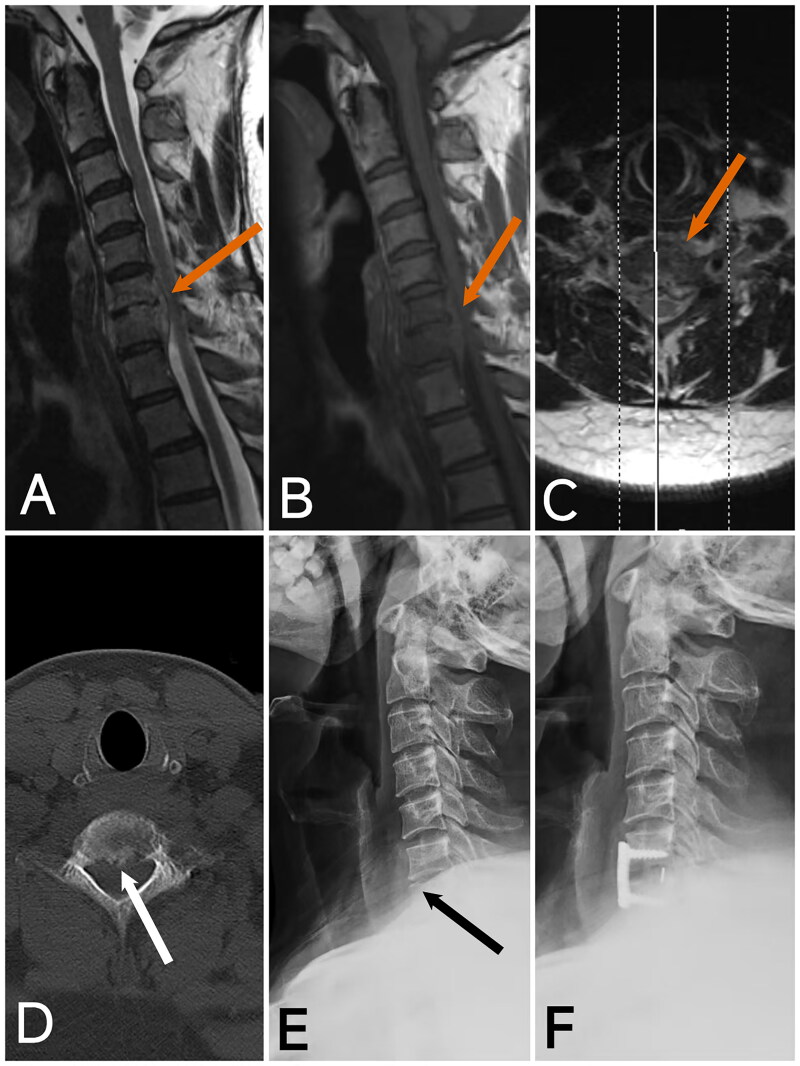
Image of case 5. A: T2-weighted imaging (T2WI); B: T1-weighted imaging (T1WI); C: Axial T2-weighted imaging (T2WI) shows an epidural abscess with significant compression of the spinal cord (red arrow). D: CT reveals prevertebral bone destruction (white arrow). E: Preoperative X-ray shows loss of intervertebral height at the C6-7 level (black arrow). F: Postoperative X-ray at 3 months demonstrates good positioning of the internal fixation, significant restoration of intervertebral height, and early fusion of the intervertebral bone graft.

**Figure 7.**
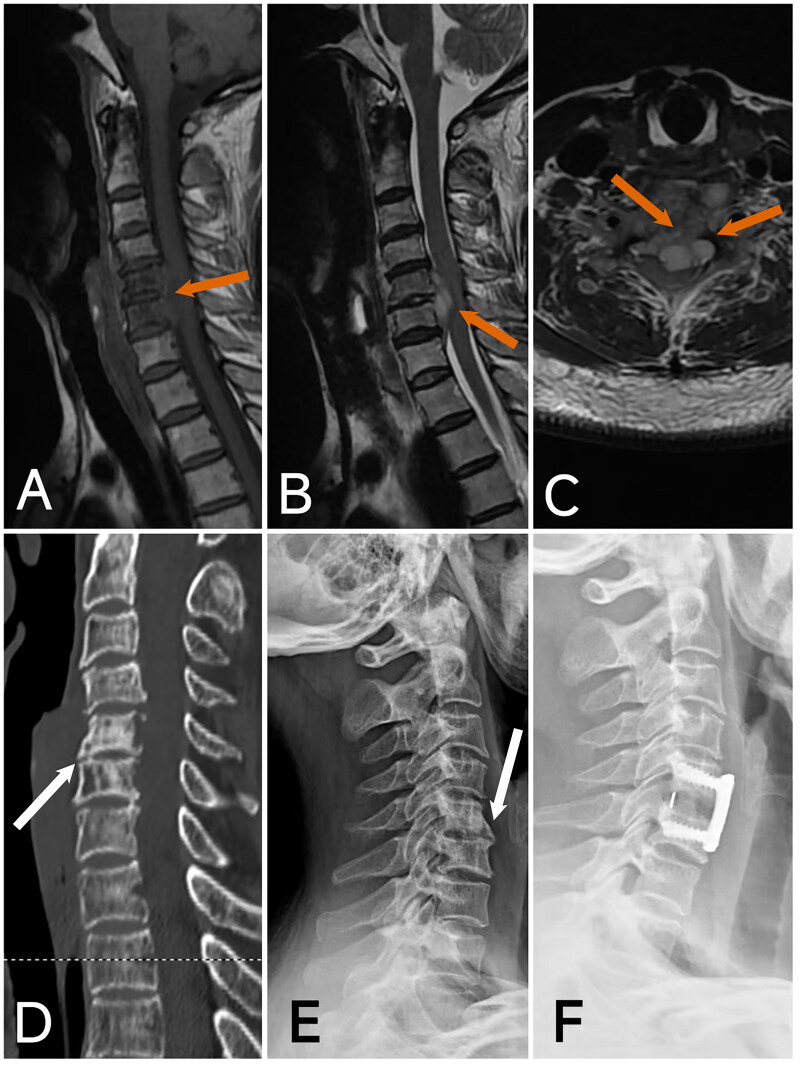
Image of case 6. Case 6: A: T1-weighted imaging (T1WI); B: T2-weighted imaging (T2WI); C: Axial T2-weighted imaging (T2WI) shows an epidural abscess at the C5-6 segment with significant spinal cord compression (red arrow). D, E: CT and X-ray reveal loss of cervical curvature, vertebral instability, mared narrowing of the C5-6 intervertebral space, and "lip-like" changes at the anterior edge of the vertebrae (white arrow). F: Postoperative X-ray at 4 months demonstrates good positioning of the internal fixation, restoration of cervical curvature, recovery of the C5-6 intervertebral height, and fusion of the intervertebral bone graft.

Preoperative cervical MRI, CT, and X-ray examinations were conducted for all patients. MRI findings revealed epidural abscesses in all patients, which exerted significant pressure on the spinal cord. CT and X-ray examinations showed relatively mild bone destruction. Patient 2 (Figure 3) was diagnosed with Klippel-Feil syndrome, and patients 3 (Figure 4), 5 (Figure 6), and 6 (Figure 7) exhibited some destruction of the anterior or posterior vertebral borders on CT. Notable intervertebral space narrowing was present in patients 2 (Figure 3), 4 (Figure 5), 5 (Figure 6) and 6(Figure 7), with patients 1 (Figure 2), 2 (Figure 3) and 6 (Figure 7) also displaying loss of intervertebral height, vertebral slippage, and instability.

**Figure 8.**
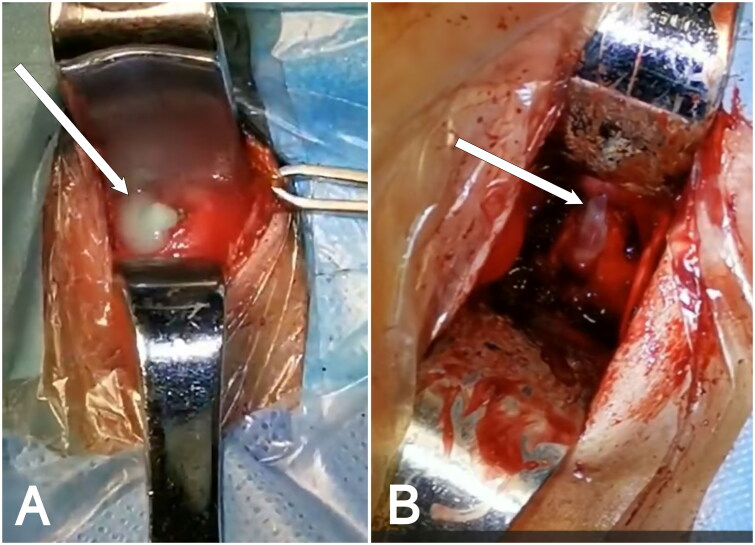
Intraoperative Pictures. A, B: Gray-white pus observed during the surgery (white arrow).

### Laboratory investigations

Elevated WBC, ESR, and CRP levels were observed in all patients. All patients tested positive for RBPT, and STAT test titres exceeded 1:100 (4-fold dilution). Blood cultures from the febrile patients were negative (Tables 2, 3). Postoperative cultures of purulent and necrotic tissues were also negative, and pathological examination revealed chronic purulent inflammation and granulation tissue proliferation.

**Table 2.** Median and interquartile range of VAS, JOA, NDI, WBC, CRP and ESR at each time point.

Time Point	VAS Score M(IQR)	JOA Score M(IQR)	NDI Score M(IQR)	WBC (10^9/L) M(IQR)	CRP (mg/L) M(IQR)	ESR (mm/H) M(IQR)
Preoperative	6(6-6)	6(4.25-10.75)	29.5(25-31.75)	6.535(4.76-9.93)	57.7(14.65-106.15)	46.5(28.5-55.5)
At Discharge	2(2-2.75)	14(12.25-15)	15(13.5-15.75)	4.715(4.08-7.135)	20.45(11.75-65.75)	38.5(8.75-59.25)
3-Month Follow-up	1(1-1)	16.5(16-17)	6(4.5-7.5)	4.57(3.0875-5.475)	6.55(2.9825-7.05)	3.5(2.25-4.75)
Last Follow-up	0(0-0.75)	17(17-17)	4.5(2.5-5.75)	4.89(3.69-5.19)	5.2(2.265-6.275)	4(2.5-4)

**Table 3.** Comparison of STAT and RBPT preoperative and postoperative.

	RBPT		STAT			
Time Point	Positive	Negative	1:50	1:100	1:200	1:400
Preoperative	6	0	0	2	1	3
3-Month Follow-up	3	3	2	1	0	0
Last Follow-up	0	6	0	0	0	0

RBPT: Positive and Negative counts as recorded for each time point.

STAT: Shows the number of cases with specific dilution results for each time point.

### Surgical outcomes

All patients underwent ACDF. The procedures for infection debridement, decompression, and bone graft fusion were performed successfully. At follow-up, all patients were confirmed to have stable intervertebral bone fusion by CT or X-ray, with a median fusion time of 4.0 (IQR, 3.0–4.25) months. No associated complications were observed. Follow-up evaluations demonstrated significant improvements in ASIA scores: preoperative scores were B/C, and postoperative scores were D/E. Among them, three patients improved from grade C preoperatively to grade E at the final follow-up, two patients with grade B preoperatively improved to grade D and grade E at the final follow-up, respectively, and one patient improved from grade C preoperatively to grade D at the final follow-up. The median VAS score for pain decreased from 6.0 (IQR, 6.0–6.75) preoperatively to 0.0 (IQR, 0.0–0.75) at the final follow-up. The median JOA score increased from 6.0 (IQR, 4.25–10.75) preoperatively to 17.0 (IQR, 17.0–17.0) at the final follow-up. The median NDI improved from 29.5 (IQR, 25.0–31.75) preoperatively to 4.5 (IQR, 2.5–5.75) at the final follow-up. (Tables 2, 4)

**Table 4.** Comparison of ASIA classification preoperative and postoperative.

Comparison of ASIA Classification Preoperative and Postoperative				
No.	Preoperative	At Discharge	3-Month Follow-up	Last Follow-up
1	B	D	E	E
2	B	D	D	D
3	C	D	D	D
4	C	C	D	E
5	C	D	E	E
6	C	E	E	E

ASIA Classification:.

B: Incomplete motor and sensory loss below the neurological level.

C: Incomplete motor or sensory loss below the neurological level.

D: Incomplete motor or sensory function below the neurological level with more than half of the muscles below the level having a muscle strength of 3 or more.

E: Normal motor and sensory function.

Additional Notes:.

No. 2: Left upper limb residual symptoms with muscle strength approximately grade 3.

No. 3: Residual bladder dysfunction.

### Infection control and recurrence

Effective control of the infection was achieved through postoperative antibiotic therapy. Regular follow-up examinations revealed no recurrence of *Brucella* infection in any patient. Antibiotic treatment was continued until CRP and ESR levels normalized and both RBPT and STAT tests were negative. (Tables 2, 3)

## Discussion

Early ACDF surgery can relieve spinal cord compression and improve acute spinal cord dysfunction caused by cervical *brucellosis*. This may offer a valuable clinical strategy for managing the rare clinical condition of acute spinal cord impairment resulting from *brucellar* cervical spondylitis. Acute spinal cord impairment due to cervical *Brucella* infection is exceedingly rare in the existing literature, primarily due to the scarcity of cases and the non-specific clinical manifestations of *Brucella* infection. Common symptoms of human *brucellosis* include fever, chills, night sweats, drowsiness, myalgia, and arthralgia [25], while patients with acute spinal cord impairment may also present with sudden-onset neurological symptoms. In our study, all patients had a prolonged history of neck pain prior to the onset of acute spinal cord impairment, yet only two patients exhibited fever. This suggests that cervical *brucellosis* may possess significant latent characteristics, potentially contributing to the difficulty in early diagnosis and progression to spinal cord impairment. Therefore, early diagnosis and timely treatment may be crucial in preventing severe damage in patients with neck pain in *Brucella*-endemic areas, particularly those with a history of contact with cattle or sheep [26].

Imaging studies play a pivotal role in the diagnosis and management of cervical *brucellosis*. X-rays and CT scans can identify characteristic features of *brucellosis* [27], such as spinal instability, loss of curvature, intervertebral space narrowing, vertebral height loss, vertebral edge destruction, and bone spur formation. CT imaging can provide detailed visualization of anterior and posterior vertebral edge destruction and show the extent of paravertebral and epidural abscesses. Additionally, X-rays and CT scans are essential for detecting congenital bony abnormalities, which can aid in diagnosing Klippel-Feil syndrome, as observed in case 2. (Figure 3) Klippel-Feil syndrome may lead to cervical canal stenosis, exacerbating the patient’s symptoms and prognosis. MRI remains the most critical imaging tool for diagnosing and treating *brucellar* spondylitis, offering detailed insights into epidural and paravertebral abscesses [2,28]. Our case analysis revealed that all patients with acute cervical spinal cord impairment had severe epidural abscesses compressing the spinal cord. This underscores the importance of early detection and timely intervention for epidural abscesses to potentially prevent severe neurological impairment.

In laboratory tests, although all patients tested positive for the STAT and the RBPT, cultures from purulent material and blood cultures from the two febrile patients were negative. This highlights the significance of STAT and RBPT in the diagnosis of *brucellosis*. Furthermore, changes in white blood cell count alone were insufficient to reflect the infection status, with ESR and CRP proving more effective for monitoring infection and patient recovery stages.

Regarding treatment, all cases underwent ACDF early in the course of spinal cord impairment, achieving favorable outcomes. Preoperative, discharge, 3-month postoperative, and final follow-up ASIA scores, along with VAS, JOA, and NDI scores, showed favorable improvement postoperatively. Surgery effectively relieved spinal cord compression, stabilized the spine, and restored spinal curvature. Therefore, based on the above results, early ACDF surgery appears to be a promising option for the treatment of acute spinal cord impairment caused by *brucellar* cervical spondylitis. Notably, in our cohort of six patients, four received surgeries with the use of a fusion cage, while two did not, which could potentially affect the clinical outcomes differently. Additionally, appropriate rehabilitation therapy plays a positive role in the recovery of spinal cord impairment [29]. However, more intensive rehabilitation interventions were not employed in our patients, which may have had an impact on their ultimate clinical prognosis. Postoperative antibiotic therapy was also crucial. According to the World Health Organization (WHO) recommendations, a combination of doxycycline (100 mg, twice daily), rifampin (600 mg daily), and streptomycin (1 g daily for 21 days) is effective [13,30,31]. Studies indicate that combined doxycycline and rifampin therapy is more effective for patients at risk of recurrence [13,32], with at least three months of long-term antibiotic treatment considered vital to prevent relapse [30]. Our antibiotic regimen included rifampin (600 mg daily) and doxycycline (200 mg daily), with continuous monitoring of STAT, RBPT, ESR, and CRP levels to determine the appropriate time for discontinuation. Although adverse reactions to rifampin and doxycycline have been reported, our patients did not experience any drug-related side effects during the treatment period.

## Limitations and challenges

The infrequency of cervical spinal cord impairment caused by *Brucella* infection limits the sample size of this study. Despite the favorable outcomes observed with surgical treatment and follow-up, larger-scale clinical trials are necessary to confirm the applicability of these results more broadly. Additionally, given the small sample size, we were unable to clearly determine the impact of specific surgical differences and postoperative rehabilitation variations on clinical outcomes in this condition. Larger sample sizes and more detailed cohort studies are needed to confirm these effects. Furthermore, the diagnosis and management of *Brucella* infections require consideration of regional and epidemiological factors to improve the rates of early diagnosis and treatment efficacy.

## Future directions

Future studies should prioritize the development of advanced early diagnostic techniques and the optimization of treatment strategies for cervical conditions resulting from *Brucella* infection. Additionally, further exploration into the effects of different treatment modalities on the prognosis of spinal cord impairment is needed to provide more accurate and effective treatment options for affected patients.

## Conclusion

Acute spinal cord impairment due to cervical *Brucella* spondylitis is a rare and serious condition with latent and non-specific characteristics that complicate early diagnosis. Current blood and pus culture methods have limitations in diagnosing this condition, highlighting the need for more sensitive diagnostic methods such as STAT and RBPT. MRI is essential for accurately assessing spinal cord compression. Our research indicates that early ACDF surgery is effective in relieving spinal cord compression, improving neurological symptoms, and restoring vertebral stability, which may make it one of the beneficial treatment options for this clinical situation. Long-term drug therapy is the recommended standard treatment for *brucellosis* and has distinct advantages in preventing recurrence. Thus, combining early ACDF surgery with long-term pharmacological therapy may provide a favorable treatment option for acute spinal cord impairment resulting from *brucellar* cervical infection.

## Data Availability

If the requirements are reasonable, the relevant data can be obtained from the corresponding author.
